# Prognostic scores in brain metastases from breast cancer

**DOI:** 10.1186/1471-2407-9-105

**Published:** 2009-04-07

**Authors:** Carsten Nieder, Kirsten Marienhagen, Sabrina T Astner, Michael Molls

**Affiliations:** 1 Radiation Oncology Unit, Nordlandssykehuset HF, 8092 Bodø, Norway; 2 Institute of Clinical Medicine, Faculty of Medicine, University of Tromsø, Tromsø, Norway; 3 Department of Oncology, University Hospital of North Norway, Tromsø, Norway; 4 Department of Radiation Oncology, Klinikum rechts der Isar der Technischen Universität München, 81675 Munich, Germany

## Abstract

**Background:**

Prognostic scores might be useful tools both in clinical practice and clinical trials, where they can be used as stratification parameter. The available scores for patients with brain metastases have never been tested specifically in patients with primary breast cancer. It is therefore unknown which score is most appropriate for these patients.

**Methods:**

Five previously published prognostic scores were evaluated in a group of 83 patients with brain metastases from breast cancer. All patients had been treated with whole-brain radiotherapy with or without radiosurgery or surgical resection. In addition, it was tested whether the parameters that form the basis of these scores actually have a prognostic impact in this biologically distinct group of brain metastases patients.

**Results:**

The scores that performed best were the recursive partitioning analysis (RPA) classes and the score index for radiosurgery (SIR). However, disagreement between the parameters that form the basis of these scores and those that determine survival in the present group of patients and many reported data from the literature on brain metastases from breast cancer was found. With the four statistically significant prognostic factors identified here, a 3-tiered score can be created that performs slightly better than RPA and SIR. In addition, a 4-tiered score is also possible, which performs better than the three previous 4-tiered scores, incl. graded prognostic assessment (GPA) score and basic score for brain metastases (BSBM).

**Conclusion:**

A variety of prognostic models describe the survival of patients with brain metastases from breast cancer to a more or less satisfactory degree. However, the standard brain metastases scores might not fully appreciate the unique biology and time course of this disease, e.g., compared to lung cancer. It appears possible that inclusion of emerging prognostic factors will improve the results and allow for development and validation of a consensus score for broad clinical application. The model that is based on the authors own patient group, which is not large enough to fully evaluate a large number of potential prognostic factors, is meant to illustrate this point rather than to provide the definitive score.

## Background

Over the last years, increasing efforts were made to better understand prognostic factors in patients with brain metastases from breast cancer. In principle, the results of such analyses can be used to create prognostic scores, which might support decision making and treatment recommendations. The best known scores, such as the recursive partitioning analysis (RPA) classes [1], the score index for radiosurgery (SIR) [2] and the basic score for brain metastases (BSBM) [3], were created from databases containing patients with brain metastases from many different types of primary tumors. Thus, only a minority of these patients actually had primary breast cancer. The same holds true for two new scores published in 2008 [4,5]. While SIR and BSBM were derived from radiosurgery-treated patients, several studies showed that they also predict survival in patients treated with other approaches. As compared to the large group of patients with brain metastases from lung cancer, the unique biological features of breast cancer allow for therapeutic approaches that might improve the response of both extra- and intracranial disease manifestations (Trastuzumab, Lapatinib, aromatase inhibitors etc.) [6,7]. Emerging data suggest that the increasing use of these drugs might also impact on survival. In order to avoid overuse of costly treatments and the potential side effects of therapy, accurate prognostic models need to be developed. These considerations led the authors to study the usefulness of the 5 previously published prognostic scores in this particular patient population. This first head to head comparison of different survival prediction models and review of literature results on emerging prognostic factors confirmed that traditional prognostic factors such as performance status and extracranial metastases are very important, but it also suggests that better prognostic models than RPA and SIR, which performed best in the present group of patients, can be developed.

## Methods

The authors used a previously described database of female patients with brain metastases from breast cancer treated with whole-brain radiotherapy (WBRT, most often 10 fractions of 3 Gy administered via lateral opposing 6 MV photon beams that did not cover the upper cervical spine/optic nerves) with or without surgery or radiosurgery [8] for comparison of 5 prognostic scores. The patients were treated at the authors' institutions in Norway and Germany during the last 10 years. For inclusion in this study it was required that all information necessary to assign the RPA classes, i.e. the best documented and validated score, was available. Out of 99 patients in the database, 83 fulfilled this requirement and the other 16 were excluded. The score developed by Rades et al. was evaluated in all 83 patients, BSBM in 82 patients, GPA in 74 patients, and SIR in 54 patients. The patient characteristics and the percentage of missing values for lesion number and volume are shown in Table 1. Performance status prior to treatment was routinely documented in all patient charts. Information on certain primary tumor features such as grading and HER2 receptor status was available in less than 30% of the patients and therefore not included in the analyses. Hormone receptor status was known in 35 of 83 patients. Systemic treatment (chemotherapy, hormonal therapy, trastuzumab) was provided as indicated for extracranial disease manifestations, taking performance status, previous systemic therapy and organ function into consideration. At the time of analysis, 3 patients were alive (follow-up 3, 9 and 24 months, respectively). The Kaplan-Meier method was used to generate actuarial survival curves. These were compared with the log rank test. Multivariate analysis of prognostic factors was performed with the Cox proportional hazards model. A p-value < 0.05 was considered statistically significant. The analyses were performed with the SPSS statistical software.

**Table 1:** Patient characteristics, n = 83 (no male patients included)

Median age, range	57 yrs., 29–76
% age <65 years vs. ≥ 65 years	81 vs. 19
Median KPS, range	70, 30–90
% KPS 80–90 vs. 70 vs. <70	37 vs. 23 vs. 40
Median time interval*	38 mo., 1–216
% single brain metastasis, multiple, unknown	37, 46, 17
Median number of brain metastases	2
Median volume of the largest lesion, unknown	15 ml, 24%
% without extracranial metastases	22
% with controlled primary tumor	96
% with complete/incomplete surgical resection of brain metastases before WBRT	7/1
% with radiosurgery before or concomitant to WBRT	6
% with salvage surgery or radiosurgery after WBRT	5

KPS: Karnofsky performance status, WBRT: whole brain radiotherapy, * from breast cancer diagnosis to brain metastases

## Results

**Figure 1:**
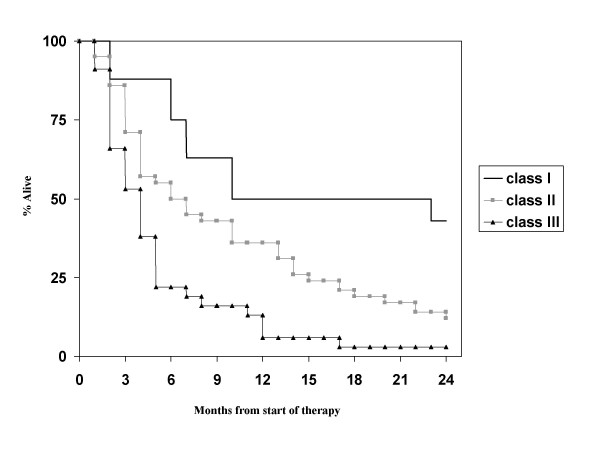
**Actuarial survival curves according to the RPA score, p < 0.01**.

**Figure 2:**
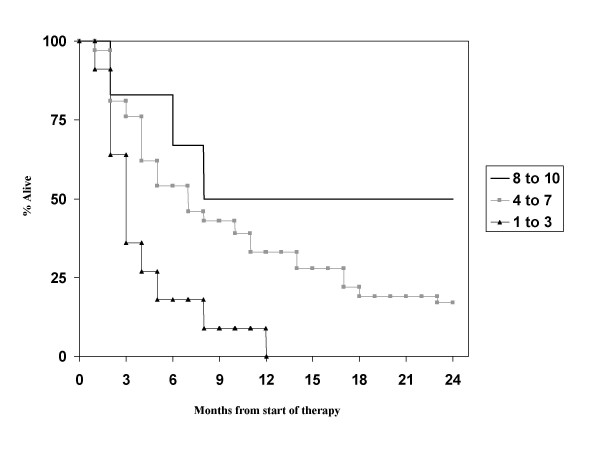
**Actuarial survival curves according to the SIR score, p < 0.05**.

Table 2 briefly recapitulates the major features of the 5 scores. First we evaluated the RPA classification. The 3 RPA classes contained 8 (10%, class I), 43 (52%, class II) and 32 (39%, class III) patients, respectively. Their median survival times were 16.0, 6.0 and 3.0 months, respectively (Figure 1). Thus, the prognostic value of the RPA classes could be confirmed. The other score with 3 prognostic classes is the SIR, which could be assigned in 54 cases. Here, the 3 classes contained 6 (11%), 37 (69%) and 11 patients (20%), respectively. As shown in Figure 2, the SIR also predicts the survival of this patient population (median 18.8, 6.0 and 2.7 months, respectively). The advantage of the SIR compared to RPA is that the tail of long-term survivors in the most unfavourable prognostic group is eliminated.

**Table 2:** The 5 prognostic scores

Score	Performance status	Age	Extracranial metastases	Primary tumor control	Interval*	Number of brain met.	Volume of brain met.
RPA 3 classes	included	included	included	included			
BSBM 4 classes	included		included	included			
SIR 3 classes	included	included	included	included		included	included
GPA 4 classes	included	included	included			included	
Rades et al. 4 classes	included	included	included		included		

* from breast cancer diagnosis to brain metastases

**Figure 3:**
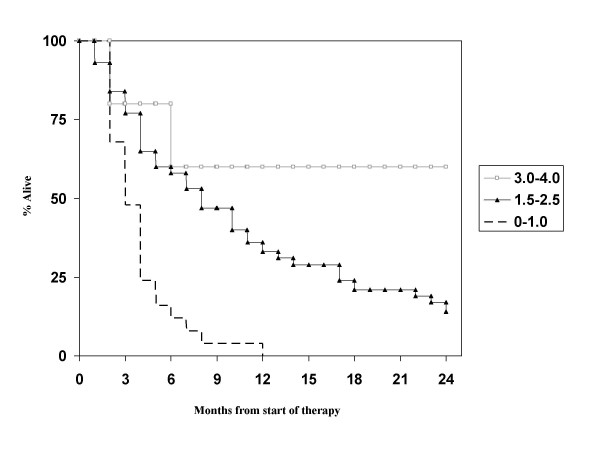
**Actuarial survival curves according to the GPA score, p > 0.1**.

**Figure 4:**
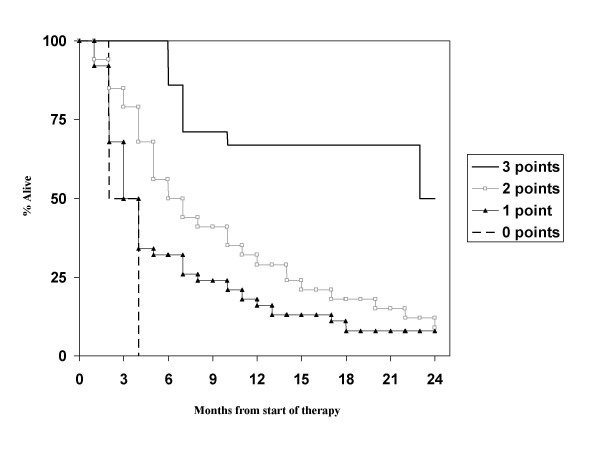
**Actuarial survival curves according to the BSBM score, p > 0.1**.

**Figure 5:**
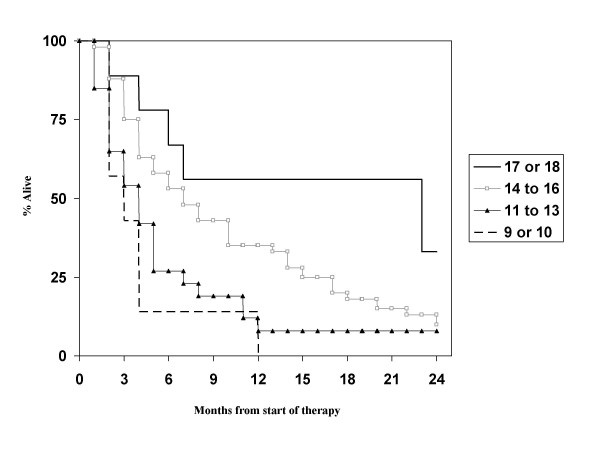
**Actuarial survival curves according to the score developed by Rades et al., p > 0.1**.

The 3 other scores contain 4 prognostic classes each. When looking at the new graded prognostic assessment (GPA) score, we discovered that only 5 of our patients belonged to the 2 most favourable GPA classes. These were therefore combined for the Kaplan-Meier analysis. The large majority belonged to the low intermediate group (n = 44, 59%) and the unfavourable group (n = 25, 34%). As shown in Figure 3, the difference between the 2 favourable classes and the low intermediate group is not statistically significant. Median survival was 55, 6.8 and 2.7 months. In the BSBM system, the difference between the 2 unfavourable classes is not statistically significant. However, the most unfavourable class contains only 2 patients and the most favourable class only 7 (Figure 4). For the score developed by Rades et al., the difference between the 2 unfavourable classes is not statistically significant either (Figure 5). Also here, only few patients belonged to the most unfavourable (n = 7, 8%) and favourable (n = 9, 11%) classes, respectively.

**Figure 6:**
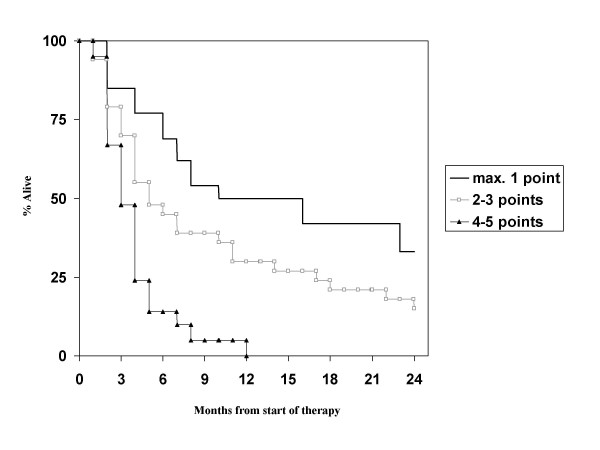
**Actuarial survival curves according to the breast cancer-specific score (3-tiered model), p < 0.01**.

**Figure 7:**
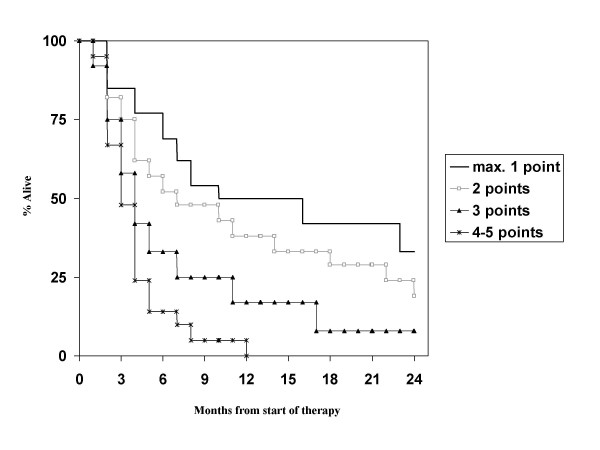
**Actuarial survival curves according to the breast cancer-specific score (4-tiered model), p < 0.05**.

Regarding the prognostic factors in our patient population, primary tumor control was not evaluated as almost all patients had controlled primaries. Age, hormone receptor status (note that information was available in only 42% of patients) and diameter of the largest lesion in the brain were not significant prognostic factors, while KPS <70, presence of extracranial metastases, presence of more than 1 brain metastasis and interval <38 months were. The strongest impact was found for KPS, while the others had similar hazard ratios in the multivariate model (Table 3). With these 4 factors, which are not completely identical to those from any of the known scores, a new score was built. Taking the different hazard ratios into account, we assigned 2 points for low KPS and 1 point each for presence of extracranial metastases, presence of more than 1 brain metastasis and interval <38 months. Overall 68 patients had information on all parameters available. Both a 3-tiered and a 4-tiered score were built and each of them performed slightly better than the previously published 3- or 4-tiered scores (Figure 6 and 7). Median survival for the groups in the 3-tiered score was 16.0, 5.5 and 2.7 months. For the 4-tiered score, 16.0, 5.5, 3.6 and 2.7 months were found. Another advantage of this score is the improved balance in patient numbers. In the 3-tiered score, the different prognostic classes contain 22, 38 and 40% of the patients (for the 4-tiered score: 19, 32, 18 and 31%). Finally, it was evaluated whether the better performance of the new score could be explained by the fact that 15 of 83 patients were excluded from the analysis (because of missing information as explained above). If the excluded patients would have been those creating problems in the other systems, e.g., the tail of long-term survivors in RPA class III, the new score would not provide a real advantage. However, it was found that the excluded patients belonged to class II (n = 11) or class III with survival <11 months (n = 4).

**Table 3:** Overview of prognostic factors in the present group of patients

Parameter	Univariate analysis (log rank test)	Multivariate analysis (Cox regression analysis)	Included in final prognostic model
Primary tumor control	not done		
Age	not significant (p = 0.09)	not significant (p > 0.01)	
Karnofsky performance status	p < 0.05	p = 0.01, hazard ratio 3.6	yes
Extracranial metastases	p < 0.05	p = 0.03, hazard ratio 2.1	yes
Interval from first cancer diagnosis to brain metastases	p < 0.05	p = 0.05, hazard ratio 1.8	yes
Number of brain metastases	p < 0.05	p = 0.05, hazard ratio 2.0	yes
Diameter of the largest brain metastasis	not significant (p > 0.1)	not included	
Hormone receptor status	not significant (p > 0.1)	not included	

## Discussion

The present analysis attempts to shed more light on the prediction of survival of patients with brain metastases from breast cancer. Experienced clinicians will be able to supplement the information provided by prognostic scores such as RPA or GPA by factors that can not easily be incorporated in such models. Examples include the number of previously administered therapies, duration of response, remaining systemic treatment options, bone marrow function, weight loss etc. Despite all efforts to predict the outcome, some patients will likely respond to treatment and do much better than predicted, while others might die from unforeseeable events such as pulmonary embolism, bowel perforation, severe infection etc. In other words, even a very advanced and accurate prognostic model remains a model, which provides less than perfect specificity and sensitivity. In an ideal world, the authors' study group would have been larger and detailed information on blood chemistry, haematology, HER2 status etc. would have been available. Taken these caveats into consideration, we can not conclude that we finally arrived at the best possible prognostic model. Our present analysis rather supports the hypothesis that a better model than the ones derived from the general brain metastases population can be developed for the patients with primary breast cancer. Without doubt, the definitive prognostic score can only be created from a very large database.

The prognostic impact of the RPA score was previously confirmed in a comparable patient population by Viani et al. [9]. In their study, median survival was 11.7, 6.2 and 3.0 months in class I, II and III, respectively. Except for class I (11.7 vs. 16.0 months), there is strong concordance with the present data. Claude et al. and Le Scodan et al. also reported a median survival of 3.0 months in RPA class III [10,11]. No separate analyses were performed for class I and II in these 2 studies. Interestingly, both studies found that lymphopenia is an important and independent predictor of survival. Lymphopenia has not been included in other analyses published so far and was not available in our patients either. Mahmoud-Ahmed et al. reported on patients treated with WBRT alone, i.e. a less favourable group. Median survival was 8.1, 6.1 and 1.7 months in the three RPA classes (p = 0.01) [12]. The other prognostic scores have not been evaluated in the previous breast cancer studies. For the present cohort of patients with brain metastases from breast cancer, the known 3-tiered scores RPA and SIR were found to reflect the prognosis, but not to a completely satisfactory degree. This might be explained by the fact that these scores originally were derived from mixed patient populations, where those with breast cancer make up just a minority. Indeed several recent analyses indicate that the prognostic factors in patients with breast primaries are not identical to those that define the known prognostic scores. Table 4 provides an overview of these analyses. The present study also arrives at prognostic factors, which are different from those that make up the known scores. By using the 4 factors that we identified, a 3-tiered score can be created, which performs slightly better than RPA and SIR and which results in almost equally large patient groups.

**Table 4:** Prognostic impact (PI) of different tumor- and patient-related parameters

	*n*	PI of hormone receptor status	PI of HER-2 status	PI of various factors (multivariate)
Claude et al. [10]	120	none	no data	performance status, lymphopenia
Bartsch et al. [16]	174	none	none	performance status, number of metast. sites
Le Scodan et al. [11]	117	receptor negative sign. worse	none	performance status, lymphopenia, hormone receptor status
Nam et al. [17]	126	receptor negative sign. worse	HER-2 negative sign. worse	number of metast. sites, age, hormone and HER-2 receptor status, leptomeningeal disease
Eichler et al. [18]	83	none	HER-2 negative sign. worse	HER-2 receptor status, number of brain metast., local disease control
Melisko et al. [19]	112	receptor negative sign. worse	none	hormone receptor status, age, performance status, stable or responding systemic disease
Harputluoglu et al. [20]	144	none	none	number of brain metast.
Park et al. [21]	125	none	HER-2 positive sign. worse	HER-2 receptor status, performance status
Altundag et al. [23]	420	receptor negative sign. worse	none	age, hormone receptor status
Wronski et al. [25]	70	receptor negative sign. worse	no data	leptomeningeal disease, combined surgery and whole brain radiotherapy
Lee et al. [22]	198	no data	no data	performance status, number of brain metast.
Viani et al. [9]	174	no data	no data	extracranial metastases, RPA class
Mahmoud-Ahmed et al. [12]	116	no data	no data	performance status
Liu et al. [24]	48	no data	no data	performance status, number of brain met.

In the recent literature a trend towards 4-tiered scores such as GPA and BSBM can be found. As previously acknowledged, the number of patients in the present study is not high enough to fully evaluate the performance of these scores in patients with breast cancer. Yet the important question is what might be the clinical impact of expanding from 3- to 4-tiered scores. Basically, the decision is whether or not to provide any local treatment beyond WBRT. Both surgery and radiosurgery were found to improve survival in prognostically better patients with brain metastases amenable to these procedures [13-15]. Given the survival curves derived from the present analysis, the 2 better prognostic groups in the 3-tiered systems in principle qualify for surgery or radiosurgery, while such treatment might not be justified for many if not most of the patients in the most unfavourable group. If one doesn't want to withheld potentially useful treatment, the group with the most unfavourable prognosis should be kept small. Moving from Radiation Therapy Oncology Group (RTOG)'s RPA to their new 4-tiered GPA score, the size of the most unfavourable class can be reduced from 39 to 34%. Using the 3- vs. 4-tiered score developed from the present database, a reduction from 40 to 31% can be achieved. The survival difference between the 2 most favourable prognostic groups in a 4-tiered system might be statistically significant, but it is not very relevant for decision making.

The challenge for the future is the validation of the current findings in a much larger database, which ideally will contain additional information on tumor biology and other host factors (receptor status, lymphopenia etc. [16-25]) and thus allow for a head to head comparison of these factors with performance status, extracranial metastases (or number of sites or disease status as suggested by different studies listed in Table 4), number of brain metastases, interval and age. The creation of such a database probably will require collaboration between several institutions.

## Conclusion

A variety of prognostic models describe the survival of patients with brain metastases from breast cancer to a more or less satisfactory degree. In the present group, the best results were obtained with the RPA and SIR score, respectively. However, the standard brain metastases scores, which often were derived from mixed patient groups (large percentage of lung cancer), might not fully appreciate the unique biology and time course of this disease. It appears possible that inclusion of emerging prognostic factors will improve the results and allow for development and validation of a consensus score for broad clinical application.

## Competing interests

The authors declare that they have no competing interests.

## Authors' contributions

CN, STA and MM participated in the design of the study, CN and STA collected patient data and follow-up information, CN carried out the statistical analysis, CN, KM and MM drafted the manuscript. All authors read and approved the final manuscript.

## Pre-publication history

The pre-publication history for this paper can be accessed here:

http://www.biomedcentral.com/1471-2407/9/105/prepub
